# Collaboration with community connectors to improve primary care access for hardly reached people: a case comparison of rural Ireland and Australia

**DOI:** 10.1186/s12913-020-4984-2

**Published:** 2020-03-06

**Authors:** Carolyn Wallace, Jane Farmer, Carolynne White, Anthony McCosker

**Affiliations:** 1 Swinburne University of Technology, Mail Services Unit, H25, PO Box 218, Hawthorn, VIC 3122 Australia; 2 Mind Australia, PO Box 592, Heidelberg, VIC 3084 Australia; 3 Swinburne University of Technology, Mail Services Unit, H31, PO Box 218, Hawthorn, VIC 3122 Australia

**Keywords:** Primary health care, Access to health care, Boundary spanning, Community connectors, Community health workers

## Abstract

**Background:**

This study presents a way for health services to improve service access for hardly reached people through an exploration of how staff can find and collaborate with citizens (referred to as connectors) who span socio-cultural boundaries in their community. The study explored the local socio-cultural contexts of connectors’ boundary spanning activities and if they are health related; boundary spanning occurring between connectors and health professionals at the interface of health systems and community; and the opportunities and barriers to actively seeking out and collaborating with community connectors to access marginalised and hardly reached people.

**Methods:**

We conducted a qualitative case comparison from rural Ireland and Australia. Following purposive snow-ball sampling techniques to recruit participants, semi-structured interviews were conducted with 34 community informants, 21 healthcare staff and 32 connectors. Transcripts were coded and analysed using an inductive approach to ascertain categories and overall themes.

**Results:**

We found a diverse sample of connectors relating to heterogenous, small and locally distinct groups of hardly reached people. Overall 26 connectors were active at the interface between health services and the community, with variation in how this occurred between cases. The majority (21) described one or more health related activities with hardly reached people. All connectors expressed a willingness to develop a relationship with local health services on issues they identified as relevant. Barriers to collaborations between connectors and health services related to bureaucracy, workload, and burnout.

**Conclusions:**

Collaborating with connectors has potential as one strategy to improve access to health services for hardly reached people. To enact this, health staff need to identify local socio-cultural boundaries and associated connectors, facilitate two-way connections at the boundary between health services and community and enable collaboration by attending to activities in the community, at the interface between health services and community, and within the health system.

## Background

The purpose of this study is to investigate how community members can support greater access to primary healthcare by examining the behaviours and interactions of existing emergent connectors, understanding what they do and considering how their work could be extended or supported by health services. To verify that this phenomenon is widespread, case studies were conducted two countries. The paper presents the idea that citizens that already connect with hardly reached people to improve social inclusion in communities, could be potential collaborators with health services in facilitating greater accessibility to primary care services. We refer to these people as community connectors (connectors) defined as socially engaged citizens who facilitate flows of information, relationships and access to resources between different and disconnected parts of the community [1, 2]. The paper explores how connectors operate in two countries, and how their work could develop as one strategy to generate more equitable health service access.

The World Health Organisation (WHO) provides global leadership to reduce health inequalities as affirmed in the *Declaration of Astana* [3]. It recognises that access to primary care, the “first contact with health services”, is a foundational requirement for good health and that citizens require the resources to enable such contact. Once this capability to approach services is attained, citizens can benefit from primary health services across the lifespan including prevention, screenings, and management of noncommunicable and communicable diseases [3].

Capability to reach that first contact implies holding a range of social and cultural resources that enable a person to make active approaches or to accept proactively offered services. Actual utilisation requires that services are approachable, acceptable, available, accessible and affordable, where approachability includes outreach activities and making the service and its offerings known to a range of demographic groups; and availability includes geographic location, accommodation, hours, and appointment mechanisms [4]. Discussions of health service accessibility often focus on individual attributes and system barriers [5–7], rather than considering how the abilities of potential users influence service accessibility [8]. The former approach misses the idea that making first contact is fundamental and influenced by socio-cultural factors. The latter approach, highlights the socio-cultural dimension by conceptualising service access as the ‘fit’ between the user and the system, with access as a process of seeking and obtaining care [4, 9]. Levesque et al. [4] encapsulate this in their framework of access to health care which outlines the capabilities of individuals and the dimensions of organisational accessibility that interact at the interface between the user and the service to impact positively or negatively on access to services.

Even in high income countries, with apparently almost universal health coverage [10], citizens can be deterred from interacting with health services because they do not feel “socio-culturally comfortable” with them [11, 12]. In some cases, barriers are more obvious and thus more comprehensible, such as for Indigenous people who may find mainstream services insufficiently culturally secure [13]; or for people from minority racial and ethnic groups faced with language barriers and beliefs and practices incongruous to their own [14, 15]. However, other groups termed “marginalised”, “hard-to-reach” or “underserved” also face “layers of vulnerability” [16] and cultural barriers due to social classifications such as disadvantaged status, gender and sexuality [17–19]. Rather than considering these citizens as “hard to reach”, this study adopts the idea of “hardly reached” [20], placing the onus on service providers to consider the unseen socio-cultural boundaries which lead to services being hardly reached by some citizens.

This study’s exploration of connectors in Australian and Irish rural sites moves beyond the commonly applied classifications for marginalised and hardly reached groups, to consider more localised and contextualised boundaries. The term “boundaries” refers to the “markers of difference” [21] emergent through cognitively and socially framed processes that define what is inside and what is outside [22]. Socio-cultural boundaries define the diverse cultures, each bounded by shared beliefs, values and norms, that exist in a geographically defined community [23, 24]. Interactions of interrelated systems of formal and informal socio-political groups in a community [13, 25] lead to processes of social stratification where mainstream behaviours and practices typify the “dominant” groups as distinguished from stigmatised “nondominant” groups [19, 26]. At a community level, social status is represented through locally understood cultural symbols such as participation or roles in volunteering activities, sporting and social events [18, 27, 28], and experienced in ways that are gendered, economic and moral [27]. Further markers of difference result from demographic change affecting contemporary rural communities in both Australia and Ireland with internal migration for housing affordability (welfare migration) or life style factors (tree change) where boundaries are drawn between locals with in depth experience of the local culture [29, 30] and recently arrived “newcomers” viewed as “outsiders” [26].

Those defined by local socio-cultural boundaries as nondominant, do not share the local cultural toolkit and are thus marginalised, with restricted access to social and economic capital [23, 25, 26, 31, 32]. The characteristics and circumstances of citizens classed as nondominant may limit their ability to access services, but there is also the issue of ‘fit’ between the user and the system [4, 8]. Marginalised citizens from nondominant groups are often hardly reached by primary care services because the mainstream health system caters for the needs of dominant groups but fails to adapt its approachability, acceptability or availability in response to the status disparities and differences within a community arising from local socio-cultural boundaries [18, 28, 33].

The challenges associated with enabling marginalised people equitable access to care is an enduring global and national health policy issue. The WHO provides an international framework for integrated, people-centred health services using a community based approach to concentrate on improving access for “underserved and marginalised” populations [34]. In Australia, the Long Term National Health Plan sets out an approach to make primary health care more patient focused and accessible [35], supported through a national system of Primary Health Networks and state health policies and programs such as the Victorian Government’s place based approaches partnering with local communities to identify and address access barriers [36]. The Irish health system is reorienting from a hospital centric model to a universal single tier health system. This includes a new focus on empowering people and communities with explicit support to leverage the expertise of health and social care staff working in community settings and partnerships with local government for community development [37, 38].

As a result of such policy commitments, in different countries, a range of types of health workers have emerged, targeted at improving peoples’ capacity for initial engagement with health services, and thereafter to leverage more equitable access to primary prevention and care services A widely deployed strategy is harnessing close-to-community (CTC) providers, local citizens recruited to act as a bridge between communities and health systems [39]. The most widely recognised of these roles is Community Health Workers (CHWs), defined as members of the communities where they work, selected and answerable to the communities, supported by the health system but not necessarily a part of it, with shorter training than professional workers [40]. While CHWs tend to be associated with low or middle income countries [39, 41], high income countries are increasingly realising the benefits of such bridging roles for reaching ethnically and racially diverse groups [15, 42]. CHWs are one among several types of CTC bridging roles, also including navigators, lay workers and peer supporters [43].

Despite deployment of CTC workers and widespread commitments to implementing WHO principles of supporting people to access primary care, hardly reached groups and associated health inequalities persist [44]. In the study reported here, we were first approached by an Australian rural community health service seeking assistance to span socio-cultural boundaries between local health services and hardly reached community members, to improve access and engagement. In responding to this challenge, our study moves beyond a focus on formalised CTC roles to explore the potential of already emergent citizens (connectors) who naturally catalyse connections between community members, including involving hardly reached people. Connectors with the cultural capabilities to access health services and the motivation to link hardly reached people with resources, including access to health services, are a potential bridge between health services and hardly reached people. We conceptualised connectors as “boundary spanners”, a term originating in business and management literature [45] and since more widely adopted in social science [46] and organisational studies of health and education [47, 48], to describe people who act as the links between a unit and its environment for purposes of information exchange, access to resources, innovation and group representation [45]. We used the term “community connectors” (connectors) which we found citizens liked more than the term “boundary spanners”. We were particularly interested in connectors’ interest and capacity to span boundaries at the interface of health services and community, as expected of citizens in formal CTC roles working with health care services. Undertaking research at two sites enabled exploration of how collaboration involving connectors and health services occurred at one site where boundary spanning was already identified by the health service and another site where it had not been considered. Sites at two countries enabled exploration of contextual differences such as health system variation.

Working with local citizens in CTC bridging roles is an emergent strategy in high income countries, but there is limited research on the establishment, effectiveness and enablers for such roles [15, 42], limited research in rural settings and even less relating to collaborating with connectors on their terms as opposed to recruiting them to formalised CTC roles. It is significant, therefore, to understand how the idea of connectors is received and understood by health services and communities in different high-income country settings, allowing for consideration of this strategy for enabling accessibility in high income countries. The research questions were: What are the local socio-cultural contexts of connectors’ boundary spanning activities and to what extent are they health related? What is the nature of boundary spanning between connectors and health professionals? What are the opportunities and barriers to actively seek out and collaborate with connectors to access hardly reached people?

## Method

### Study design

A comparative qualitative research study design was used, with instrumental in-depth case studies in Victoria, Australia and Ireland, to enable similarities and differences to emerge [49]. This strategy enabled understanding of the factors and context that shaped the boundary spanning of connectors, while international, comparison allowed us to search for constant factors and concepts that “travel” beyond a single social and policy context [50, 51]. Ethics approval was granted by the Swinburne University of Technology, Swinburne University Human Research Ethics Committee (SUHREC), SHR Project. 2017/110, with a formal amendment approved to cover the Irish case which was facilitated through a Local Development Company (LDC) (i.e. a non-health service body).

### Case study sites

#### Australia

Australian healthcare is jointly funded by Commonwealth and State governments, with service delivery primarily a State responsibility. The Australian site was in the state of Victoria with one of the 81 publicly funded health services, all of which are governed by local boards intended to reflect community diversity. The service provides primary health care, acute care and residential aged care, with campuses in three towns. The study took place in the largest of the three towns, with populations of 2316 and 541 [52]. The area is characterised by its rural nature and distance from larger centres. Both towns have a lower number of overseas born residents (16.8 and 14.4%) compared to the Australian figure of 33.3% [53]. The majority of residents have multi-generational or long-term connections with the town, alongside a more recent influx of residents seeking either the rural lifestyle (tree changers) or more affordable housing (welfare immigrants).

#### Ireland

In contrast to Australia, Irish health services are not locally governed. The Irish public health system is managed centrally by the Health Services Executive (HSE). Services are delivered through seven hospital groups and nine community healthcare organisations. Government strategy encourages governmental health entities to develop community partnerships with voluntary and other organisations to favourably influence health and wellbeing [38]. One such group of organisations are Local Development Companies (LDCs), funded by the Government to promote local economic and social participation. In the absence of a comparable local health service structure in Ireland, we selected LDCs as a suitable platform with which to engage. The Chief Executive Officers (CEO) of three rural LDCs were contacted, and the study proceeded with the LDC that indicated the study aligned with organisational strategy. This LDC covers a rural area in the south east of Ireland. An amendment to the SUHREC approved ethics statement SHR Project. 2017/110 was requested, reviewed and endorsed by the LDC board. Following discussions with staff at the LDC, two towns were identified to complement current LDC wellbeing initiatives. The first town, within a 15-min drive to a large city, has rapidly expanded from a village to a commuter town. It has a population of 5080 with 23% of residents born overseas, compared to the Irish national rate of 17.3%. The second town has a population of 7963, with 16.7% of residents born overseas [54]. This town is characterised by tourism and higher than national unemployment rates due to closure of local industry.

### Participant selection

Participant sampling was purposive followed by snowball sampling [50]. Connections with the health settings provided a locally endorsed “entry point” [50] when contacting potential study participants. Community informants (*n* = 34) were identified initially by internet searches. These were people holding prominent positions in the community, including CEOs of community organisations, councillors, hotel owners, local newspaper editors and school principals, likely to have an understanding of the socio-cultural boundaries within the community. Diverse community informants were contacted to facilitate introductions to a range of connectors within the locale. Staff from health services (Australia) and the LDC (Ireland) (total *n* = 19) assisted with the identification of connectors and additional community informants. Participant sampling continued to the point of data saturation where no new or relevant information emerged from the subsequent interviews [55]. Table 1 shows the number of participants in each case and key demographic features of the connectors.

**Table 1:** Participants

	Australia	Ireland	TOTAL
**Community informants**- prominent local people likely to identify socio-cultural boundaries and connectors	17	17	**34**
**Staff** -from health services and LDC	8	11	**19**
**Connectors** -spanning a range of socio-cultural boundaries	15	17	**32**
**Connector age**			
35–65	11	12	23
66+	4	5	9
**Connector gender**			
Female	13	11	24
Male	2	6	8
**Connector employment status**			
Employed	10	10	20
Retired	5	3	8
Unemployed/sickness benefit	0	3	3
Home duties	0	1	1
**Connector length of time in community**			
Entire life (with some years away for work or study)	13	8	21
More than 15 years	0	7	7
Less than 15 years	2	2	4

Staff and community informants were provided with an introductory text and asked to invite the connectors they identified, to participate. Connectors who agreed to interview were asked, in turn, to identify and contact other connectors. Ultimately identified connectors (*n* = 32) included a diverse sample of local socio-cultural contexts of connectors’ boundary spanning (Table 2).

**Table 2:** Boundaries for hardly reached people

Boundary Category	Australia	Ireland	Total
Age	6	10	16
Family structure	5	6	11
Income	7	1	8
Gender	5	3	8
Nondominant group/social status	5	3	8
Immigrant, non- English speaking background	0	6	6
New to town, from another part of the country	2	3	5
Mental health problems	3	4	7
Disability	0	3	3
Health concerns	2	1	3
Transiency or homelessness	2	1	3

### Data collection

An ethnographic approach was adopted [56], with the researcher spending blocks of time in both communities, interacting informally with study participants and attending local events. Contextual knowledge that accrued assisted with rapport building in interviews and in probing experiences of socio-cultural boundaries and relationships with local organisations [50]. Data collection occurred over three-months at each site, in 2017 (Australia) and 2018 (Ireland).

Interviews were recorded with consent and transcribed; and field notes were written. Semi-structured interviews covered discussion of: community socio-cultural boundaries, access to services and community members who span boundaries to promote access and community participation. Staff were also asked about current practices of working with connectors, their views on engaging with connectors, and what activities or attitudes might support such practices. Community informants were asked about the impacts of connectors and the behaviours and characteristics that distinguished people as connectors. Connectors were asked about their motivation, their inclination to have stronger connections with health services and enablers for this activity (See Additional file 1).

### Data analysis

Data analysis was inductive and deductive [57, 58], commencing with an open coding process. Data from the case studies were imported into NVIVO software and coding was undertaken by CW, with samples reviewed by JF and AM for verification. Codes were compared with existing evidence [59, 60] from a prior scoping review [43] and codes relating to the benefits of boundary spanning were added. Additional insights from coding the Irish transcripts were used to review and modify coding of the first case. Overall themes were refined through this iterative process.

Themes were compared across the cases [49]. Contextual differences were explored through development of word tables to array features from each case [49] relating to: health actions of the connector; barriers between the health system and community; and demographics and features of the connectors (age, gender, boundary conditions they crossed, locale for their connections, and closeness to the health service).

## Findings

To understand the viability of identifying and collaborating with connectors, to improve accessibility of services, we consider: their boundary spanning with hardly reached people; their existing work related to health; and then explore factors supporting or hindering collaborations between health services and connectors. We draw on quotes from Ireland and Australia, to illustrate. All participants are referred to by pseudonym.

### Boundary spanning in the community

Connectors in both countries described socio-cultural boundaries leading to marginalisation of some people (Table 2). Hardly reached people experiencing effects of socio-cultural boundaries were referred to in terms such as *“people who don’t have the wherewithal”* (Maire, connector, Ire) or *“edge groups”* (Heather, connector, Aus). Individual health or living conditions also acted as boundaries to participation in community life. In most instances, people with whom connectors interacted were perceived as being hardly reached based on more than one category such as young unemployed men who attended a community garden “*lads that are on community service that get in trouble with the law, come here and they do their hours” (Fergal, connector, Ire)* or single mothers new to the town where “*there’s nothing worse than being a single mother, in a community where you know nobody and the baby is crying and you don’t know what to do” (Maire, connector, Ire).*

Connectors’ membership of multiple dominant/mainstream groups, combined with their knowledge and interactions with hardly reached groups, positioned them as boundary spanners. Connectors reported responding to local needs of longstanding or emergent groups or the isolation of individuals as illustrated:

*We are beginning to get more and more people squatting for lack of a better term, or being pushed out of everywhere that they’ve been pushed out of, and we’re getting them with huge issues down the lake (Susie, connector, Aus).*

*A lot of people have just moved in, new to the area, first time mum. You get talking to them and you realise they’re kind of lonely, or you realise that you should check in (Frances, connector, Ire).*

Regardless of the boundary, connectors reported noticing how this limited participation in community life.

*[retired] men are not as inclined to be involved in any of those [social] things. And the mental health area comes into that as well. You have a whole cohort of people who mightn’t go as far as suicide and not in a great space (Tom, connector, Ire).*

*There are definitely families who I know feel as if they are bullied by our town and don’t know where services are (Vicky, connector, Aus).*

### Boundary spanning and health

The majority of connectors (*n* = 21) demonstrated one or more health-related activities in their boundary spanning. Table 3 shows these activities and number of connectors involved.

**Table 3:** Number of connectors undertaking health activities

Health activity	Australia	Ireland	TOTAL
Service navigation or referral	6	5	**11**
Provide emotional support to people with health problems	5	3	**8**
Facilitate access to health education and information	4	4	**8**
Initiate new health activity	1	5	**6**
Contribute to service planning	2	1	**3**

Service navigation or referral was the most common activity overall, as typified here with supporting an older man with depression,

*I got him a counsellor and I said a few other little bits, kind of advising him…I said I’ll call you at home on Sunday and we’ll have a chat again. So I called Sunday evening (Finbarr, connector, Ire).*

Connectors enabled health promoting opportunities in settings where they had influence. In most instances, this was through accommodating health activities or information into community programs or interactions. A noticeable difference between the country settings was the number of connectors initiating a new activity. Only one Australian connector reported establishing new health activities which included free kick boxing classes for school children and a fitness challenge for adults. Five connectors in Ireland reported initiating new health activities: a school garden; a suicide prevention and support group; fitness activities for older adults; and a wellbeing program for people with mental health issues.

In addition to heath related activities, 25 of the connectors (*n* = 11 Aus, *n* = 14 Ire) were involved in activities related to the social determinants of health, including strengthening social and community networks and enabling improvements to people’s living and working conditions. The focus of social inclusion connections was responsive to local boundaries. In Australia, the most commonly observed boundaries were socio-economic circumstances of long-term residents and welfare migrants while one of the towns in Ireland had recent immigrants from over 40 nationalities. Connectors reported how their boundary spanning had positive impacts for hardly reached people including improvements to: social connections; mental and physical health; employment; financial situation; housing and access to health services. However, not all interactions with hardly reached people led to an ongoing connection with a health service, as in this example,

*I said did you hear about the walking group? And she’s new to the area… I said sure I’m passing, I’ll pick you up…. She stayed for a few weeks, and it wasn’t for her. But that’s not the point. The point is she gave it a go, and she might have another person to say hello to (Breda, connector, Ire).*

The health activities of connectors demonstrated boundary spanning at the interface of the health services and community. In most instances contact was initiated by connectors rather than local health services (Table 4) such as a connector from a men’s shed in Ireland who worked with other shed members to organise a men’s health day,

*we invited the different people in. The Irish Heart Foundation, they came in. We had the podiatrists checking their feet. There were several different nurses from the HSE that came in. There was a huge crowd (Leo, connector, Ire).*

However, some had formed a relationship with staff at the local health service (Table 4), either through seeking them out or because a staff member had contacted them in their role as a potential connector to a hardly reached person or group. The latter is illustrated in one example where a connector established an alternative playgroup for the mothers she had noticed were uncomfortable at the existing playgroup. A community health manager concerned about lack of participation in their nutrition for parents’ program contacted the connector to see if the program could be delivered at the playgroup,

*So I said to [Kara], we’ve got this program that we’ve already run at the hospital. Here’s an opportunity, what do you think? Where should we do it? She said ‘the mums need that stuff, that would be great. Come to where we are, where they feel safe’ (Gerry, staff, Aus).*

**Table 4:** Initiation of health activities and connections with health services

		Australia	Ireland	TOTAL
**Instances of initiation of contact between connector and health service**	Connector initiated contact with health services	10	9	19
	Health service initiated contact with connector	5	3	8
**Connectors with connections with services**	Connectors with connections with health service (as program participants and/or as connectors)	11	15	26
	Connectors with no connections with health services	4	2	6

The majority of connectors (*n* = 26) were connected in some way with a local health service meaning they were actively involved in a community program of the health service or were proactively connecting hardly reached people with health information or services (Table 4). There was variation between Ireland and Australia, in terms of the type of health organisation they connected with. All the 11 Australian connectors, who had a connection to a health service, connected with one of the campuses of the same local rural health organisation. The Irish connectors had connections with multiple diverse health services: a HSE funded community health project, a cancer support organisation, community mental health services and HSE staff with regional health promotion roles. There were no connections with the HSE primary care clinic in either town.

In both countries, connectors with no connections at all to the health system indicated they would welcome the opportunity to establish local connections and all the connectors with existing connections indicated they would like to strengthen them, to focus on issues that were of interest to them and the people they connect with.

### Enablers and barriers to collaboration

In both countries we found connectors to be a naturally occurring phenomenon and resource, spanning socio-cultural boundaries to increase people’s participation in community life. Given the majority of connectors also linked hardly reached people with health information or services, we sought to understand how this resource could be tapped into further and the factors that could enable or restrict collaboration between connectors and health services.

#### Enablers

While the health systems varied, the factors enabling staff to accept the idea of identifying and collaborating with connectors were similar across country jurisdictions. These are discussed below as enablers in the community, at the interface between community and health services, and for the health system.

The first enabler for collaborating in the community was for staff to go to where connectors and community members interacted, rather than expecting connectors and hardly reached people to come to the health service. This capitalised on connectors’ ability to create a safe and comfortable space, in recognition that to access hardly reached people, *“We’ve got to go the other way where we actually go to them and put them in a comfortable position”* (Colin, community informant, Aus). Comments by connectors affirmed this approach such as *“the obvious thing for me would be [for them] to come in here and sit with ours”* (Ina, connector, Ire), *“come to my neighbourhood, we put a banner, a table outside with a pink ribbon on top flying high, and we will say ladies, there’s a van here, do you want to have it checked?”* (Sophia, connector, Ire).

Another enabler in the community was for health and community services to provide practical support to connector-initiated activities. This included personal support and training such as the connector leading weekly physical activity sessions with seniors benefitting from the occasional support of a trained officer *“to get us through our paces”* (Lynn, connector, Ire) or more practical supports such as financial assistance and promotion of a community transport service for people with disabilities (Ire) or purchasing gym memberships for playgroup mothers (Aus). Access to health staff and health information, and a *“consistent point of contact”* (Bec, connector, Aus) assisted connectors to share and cross promote health information among their networks.

Staff, and the connectors collaborating at the interface between health services and community, built productive relationships based on mutuality, flexibility and informality. Staff and connectors acknowledged the difference between their paid and voluntary (connector) roles. This was captured in staff comments regarding a community-based suicide prevention group where, *“we’re only professional in this part of it, they’re professionals in other parts, their levels”* (Colleen, community informant, Ire). Connectors acknowledged the expertise of staff, *“there are expert people within the HSE… so you wouldn’t interfere in their expertise”* (Peggy, connector, Ire).

Opportunities to improve collaboration between connectors and health professionals included networking events and opening up organisational training to connectors, where appropriate. Community-based training occurred in both countries, with staff, connectors and other community members participating together to develop common understandings and address a locally identified socio-cultural boundary issue. In Australia this was a seminar to understand poverty. A number of staff and connectors in Ireland participated in a suicide awareness training program, building the capacity of staff and connectors to work together on local suicide prevention groups, while one Irish community piloted a course for connectors and staff on cultural integration.

There were specific health system enablers. Instances where staff had identified connectors occurred when staff had roles that allowed for or required community interaction. Additionally, connectors, staff and community informants spoke of the value of staff with local knowledge, *“that’s your prime bridge between the community and the hospital (David, connector, Aus).* However, living locally was not necessarily a reason for staff to embrace collaborating with connectors. Staff with their own connector experiences were more appreciative and open to the value of professionals collaborating with connectors.

Staff collaboration with connectors was further enabled when there was a management and organisation culture that was supportive of such behaviour,

*I think it's in our aim or our strategic plan or whatever, is to be innovative and to try new things and we definitely have (Catrina, staff, Aus).*

Staff noted current collaborations with connectors were generally on an ad hoc basis and that changes to organisational and state policy would be required to embed such an approach,

*I think it’s just a matter of working on the system to recognize and respect the fact that this bottom-up approach, not only does it exist but it’s essential… That means investing in it the exact same way as they would need to invest in any other service. It means respecting it-backing off, it’s not to be controlled and not to be tied down (Michael, staff, Ire).*

#### Barriers

Staff and connectors experienced barriers to developing and maintaining relationships at a personal and system level, and these were also observed by community informants. These related to workload, burnout and bureaucratic processes, with variation between Ireland and Australia as to the frequency they were mentioned. In Australia, the most frequently mentioned barrier was time constraints and volunteer burnout, raised by connectors and community informants,

*I’ve had to scale back what I’m doing a little bit this term because I was having a bit of a sense, like you know, you can burn out (Kara, connector, Aus).*

In Ireland, staff cited workload pressures and role inflexibility. Bureaucratic processes were cited by connectors, community informants and staff as factors that slowed down interactions between health services and community. These referred to administrative processes such as form filling, data collection and regulatory processes as well as impersonal organisational responses as in this example,

*Do you want to write that in your research? How difficult it is for anyone to do anything because of this bureaucratic body which replies to the emails you send them without a signature (Sophia, connector, Ire).*

With respect to new or further collaboration with health services, connectors envisaged that bureaucratic requirements of health services such as client data collection could potentially compromise their informal interaction with hardly reached people as “people are very afraid to interact with services nowadays because like, what do you want that for?” (Ina, connector, Ireland). Connectors also raised potential for volunteer burnout if health service expectations did not match their availability. Staff envisaged that health services policies and funding guidelines could constrain their flexibility to prioritise identifying and engaging with connectors.

## Discussion

The study explored how connectors operate at the boundaries between the community and health services, and the potential for harnessing this activity to improve access to primary health care for hardly reached people. By applying the ideas of boundary spanning and connecting across boundaries, we investigated the socio-cultural contexts bridged by connectors, the existing health work of connectors, and enablers and barriers for staff and connectors to collaborate.

**Fig. 1:**
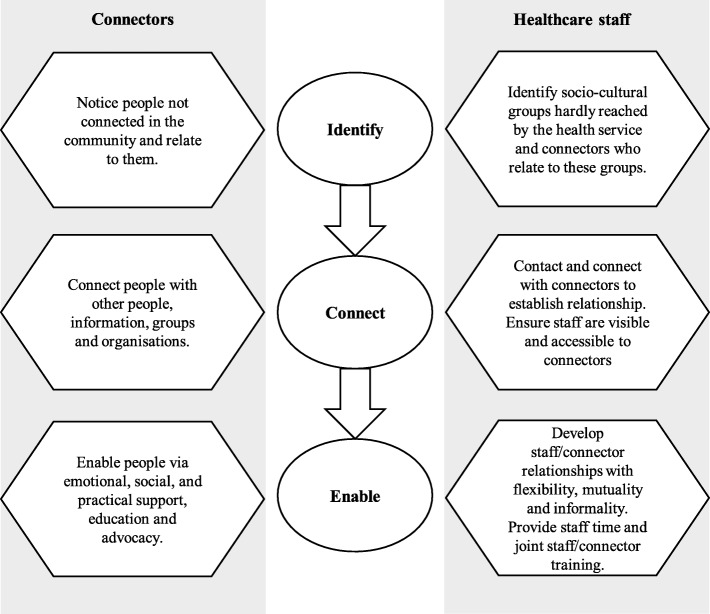
Collaborating with connectors

Here, findings are summarised and illustrated (Fig. 1) to show three key stages for health services to collaborate with connectors. The first is to *identify* socio-cultural boundaries and connectors. The second is to provide opportunities for staff and connectors to *connect.* The third is to invest in factors that *enable* collaboration with connectors while systematically addressing barriers.

Connectors were sought who were acting in a voluntary capacity, to explore the extent to which boundary spanning community members assisting with access to health, naturally occurs. The connectors fulfilled many of the functions of CTC workers, and as expected, this resembled CTC roles most strongly aligned with the community rather than the organisation, namely peer supporter, lay helper or health champion [43]. The fact that several connectors clearly articulated the difference between their community expertise and the health expertise of staff indicated appreciation that their roles differed to staff roles. It also shows that the connectors were unlikely to identify with CHW-type roles that often include delivery of simple health related service and health information sessions. Connectors are best understood as CTC collaborators able to assist with access to health services, rather than CTC workers with roles defined by healthcare staff.

This study commenced in each community by considering local boundaries and associated connectors so as to understand potential for collaborations with connectors, rather than how to recruit people to existing health programs. While access to services as the ‘fit’ between health and community systems [8] implies potential for adjustment from both sides, the reality is often still an expectation that access is about getting services to people, rather than trying to understand and respond to local socio-cultural boundaries and the expectations hardly reached people have of health services. The connectors in this study provided a safe space for health staff to enter the community milieu, with potential for staff to respond by adapting service characteristics, or their own attitudes and behaviours, to improve access. While our study has shown examples of staff making modest changes to service availability or delivery in response to requests from connectors, the reality is that funding, fragmentation of responsibility and bureaucratisation of services [61] create system boundaries that challenge the ability of local staff to make larger scale changes. Increasing pressure on health budgets, coupled with funding models based on episodes of care results in a reduction of the health promotion and community development resources [61] required for collaborating with connectors.

Because of their knowledge and relationships with both hardly reached people and staff, connectors help with the ‘fit’ between the community and health services. Connectors address health inequality gaps by supporting the capability of hardly reached people through the first three abilities of Levesque et al.’s [4] framework of access to health care: the ability to perceive a need, to seek care and to reach care. The connectors in this study did this through facilitating access to health information as well as service navigation or referral. Connectors impacted on the health of hardly reached people by noticing those who were marginalised by community socio-cultural boundaries, identifying them as having worth and identity and endeavouring to facilitate connections with other people and services while providing social and practical supports [62].

However, supporting the capability of individuals is only part of the process of accessing services, as characteristics of the local health system also impact on accessibility creating generalised institutional rules of engagement that have to be negotiated [61, 63]. These play out in the dimensions of accessibility relating to approachability, acceptability and availability [4, 9]. The health system dimensions of accessibility varied considerably between the Irish and Australian contexts. In Australia, there were smaller townships and one highly visible health service with active community partnerships, a local board of governance and a history of being supported by the community through fundraising and volunteering, all of which supported its approachability and availability. In contrast, the health service system in Ireland was more fragmented, with a range of separate services funded by the HSE, some locally based and some more centralised and consequently was less approachable and available compared to the Australian context. This could explain why there was a higher incidence of connectors initiating health activities in the Irish context, as a response to service gaps and a lack of localised service responses to local boundaries and hardly reached groups.

Despite the differences cases highlighted, the majority of connectors had some sort of connection with a health service, and all expressed a willingness for closer connections with health services on issues pertinent to their boundary spanning activity. Finding and collaborating with connectors who are attuned to local social boundaries and the existence of hardly reached people appears to be one way of achieving health policy aspirations for greater service accessibility and uptake. Connectors could be approached for partnering to help to identify and remove barriers to health care [36–38], especially barriers that are invisible to health services. Connectors are a resource ‘hiding in plain sight’, willing to collaborate with their local health services due to shared interests in supporting those hardly reached. The enablers and barriers identified in this study provide guidance to sustaining collaborations between health services and connectors as health services seek to fulfil policy expectations of accessible and responsive health services [34].

Our findings show that it takes time and local knowledge to identify connectors and that subsequent relationships between health service staff and connectors are best negotiated, not instrumentalised. While the boundary spanning of connectors assisted with the “degree of adjustment” between citizens and health resources [4], this did not occur evenly or with all connectors. This indicates that collaborating with connectors is as localised and nuanced as the nature of socio-cultural boundaries, and that collaborations with connectors may be episodic or intermittent, as determined by their availability and interests. The very localised nature of most connector interactions with hardly reached people indicates that, as boundaries change or emerge, so will potential for new relationships with connectors. Therefore, health service collaborations with connectors may occur according to a recognisable set of stages but collaborations are contingent on local boundaries and priorities, and as a result require ongoing review and adaptation.

### Limitations

Drawing on cases from two countries has allowed us to identify elements of collaboration with connectors, however conclusions are limited by the small number of cases and focus to date on rural settings only. Future research beyond the “rural locale” [13] (p 499) would provide further insights on the extent to which these elements vary in response to urban socio-cultural and policy contexts, as would studies in other high income countries. Additionally, research on the impacts of connectors for hardly reached groups, connectors, organisations and the community [43] will build the evidence base required for health services to determine the value of enabling staff collaborations with connectors. Collaborations with connectors in research processes might represent a way to ensure that such research includes the perspectives and voices of hardly reached people.

## Conclusion

This study has established that the phenomena of boundary spanning community members (connectors) collaborating with health services exists in two high income country contexts. It advances the fields of study relating to boundary spanning, CTC workers and access to health services in three ways. First, applying the concept of boundary spanning to the CTC role of connectors and to health service professionals describes what is required from staff and connectors to improve the fit between service users and the service system and thus improve access to primary healthcare. Second, our definition and study of connectors adds to CTC research in high income countries by providing evidence for a mutually rewarding collaboration between self-directed voluntary connectors and health services. Thirdly, we add to the research on health access for hardly reached groups by illustrating a way to understand and interact with the invisible and locally contextualised boundaries that exist in communities, through collaborations with connectors – people that cleverly and naturally span those boundaries.

## Supplementary information

**Additional file 1.** Interview Guides.

## Data Availability

The deidentified datasets used and analysed during the current study are available from the corresponding author on reasonable request.

## Abbreviations

CEO: Chief Executive Officer
CHW: Community Health Worker
CTC: Close-to-community
HSE: Health Services Executive
LDC: Local Development Company
