# Supplementary material for: Collaboration with community connectors to improve primary care access for hardly reached people: a case comparison of rural Ireland and Australia
Source: BMC Health Serv Res. 2020 Mar 6;20:172. doi: 10.1186/s12913-020-4984-2 (PMC7059343; doi:10.1186/s12913-020-4984-2)
Supplement: Supplementary file 1 — Additional file 1. Interview Guides. [file 12913_2020_4984_MOESM1_ESM.docx]

**Interview guides** “ **Collaboration with community connectors to improve primary care access for hardly reached people: a case comparison of rural Ireland and Australia”**

**COMMUNITY INFORMANT- INTERVIEW GUIDE**

**Introduction**

- [Partner organisation], Swinburne and the purpose of the research
- Consent
- Demographics
- Confidentiality

**Your role in the community**

- What role do you have in the community through your formal role and any others?
- How do you find out about what is happening in the community and what any issues might be?

**Networks, groups and organisations in the community**

- what are the different groups and organisations that people in this community interact with?
- what role do these have in the life of the community?
- what sort of cross over or interaction is there between these groups and organisations?

**Boundaries that separate people and stop people from accessing services or participating in community life**

- that limit access to and interaction with services?
- that limit people from fully participating in the life of the community?

**Who are the community connectors (boundary spanners)?**

- What do they do
- What impact do they have?
- How does the community respond to this or recognise this?
- Do they have any influence on how services are delivered?
- What could help them have even more impact?

**Contact with other key informants and community connectors**

- People to contact
- Information researcher can provide
- confidentiality

THANKYOU and any final questions or comments for me?

**STAFF- INTERVIEW GUIDE**

**Introduction**

- [Partner organisation], Swinburne and the purpose of the research
- Consent
- Confidentiality
- Overview of the concept of boundary spanning

**Service access**

- What services do they provide/ are they well and appropriately used by all they could help, in the community?
- What are the gaps between services and users?
- Discuss how they get people new to the community/ or people that do not use a service, and might benefit, to use/find out about services?

**Connectors and boundary spanning**

- Explain the idea of community connectors/boundary spanners, do they know any such people/organisations in the community? How do these operate? [examples]
- How was boundary spanning activity instigated, what made it work (if it did), what was the staff member’s role?
- In what ways has social media assisted in reaching harder to reach people? How do they participate in discussions with community members online? What information, if any, do they draw from online discussions and feedback about the needs of particular groups within the community?

**Barriers and enablers**

- Discuss their views on their capacity to work more formally with community connectors/boundary spanners and
- What would be needed to make this work [ for organisations, for individuals, for community capacity]

**Final points**

- Can they identify any other community connectors/boundary spanners?
- Thank them for their time and check if any more questions.

**CONNECTOR- INTERVIEW GUIDE**

**Introduction**

- [Partner organisation], Swinburne and the purpose of the research
- Consent
- Demographics
- Confidentiality

**Networks, groups and organisations in the community**

- what are the different groups and organisations that people in this community interact with?
- what role do these have in the life of the community?
- what sort of cross over or interaction is there between these groups and organisations?

**Boundaries that separate people and stop people from accessing services or participating in community life**

- that limit access to and interaction with services?
- that limit people from fully participating in the life of the community?

**Your role as a community connector**

- Why do you think you have been identified as a community connector?
- How do you find out what’s happening and keep informed?
- What do you do as a connector?
- What impact does this have:
  - Individual
  - Community
  - Organisations
  - Self
- Examples of things that did not work out?
- What role does social media or other digital technology play in this?
- What motivates you?
- What characteristics or traits do you draw on?

**Community connectors as concept and function**

- How does the community respond to what you and others like you do?
- What sort of interactions would you like to have with health services and other public entities?
- What else would you like to achieve?
- What would help you have even more impact?

**Contact with other boundary spanners**

- People to contact
- Information researcher can provide
- confidentiality

THANKYOU and any final questions or comments for me?
